# Enhancing sustainable agricultural export performance through digitalization: The distinct roles of domestic and foreign inputs

**DOI:** 10.1371/journal.pone.0339692

**Published:** 2025-12-30

**Authors:** Weifeng Huang, Yan Huang

**Affiliations:** 1 College of International Economics and Trade, Ningbo University of Finance and Economics, Ningbo, China; 2 Ningbo Philosophy and Social Science Key Research Base “Research Base on Digital Economy Innovation and Linkage with Hub Free Trade Zones”, Ningbo, China; 3 School of Statistics and Data Science, Jiangxi University of Finance and Economics, Nanchang, China; Czech University of Life Sciences Prague: Ceska Zemedelska Univerzita v Praze, CZECHIA

## Abstract

Amid the restructuring of global value chains (GVC), digital transformation has become a vital strategy for enhancing agricultural export performance and strengthening international competitiveness, while also contributing to the United Nations Sustainable Development Goals, particularly the reduction of poverty and hunger. This study utilizes the OECD Activity of Multinational Enterprises (AMNE) database and employs a two-way fixed effects model to assess the impact of agricultural digitalization inputs on export performance, measured by gross exports (E), domestic value-added in exports (DVA), and the ratio of domestic value-added in exports to gross exports (EDVAR). The results indicate that both domestic and foreign digitalization inputs significantly enhance export performance, with domestic inputs exerting a stronger effect. The heterogeneity analysis reveals that domestic inputs have a more significant impact in developing countries. Mechanism tests suggest that domestic inputs enhance production efficiency, whereas foreign inputs primarily reduce trade costs. Policy recommendations include promoting digital innovation, enhancing self-reliance and resilience, optimizing the digital trade environment, and securing adequate human capital, land, and institutional support.

## Introduction

Agriculture is the primary economic activity in many rural areas of developing countries. Improving farmers’ incomes not only enhances household livelihoods but also influences broader issues such as food security, rural development, and economic growth. Consequently, the international community has consistently prioritized income growth among farmers, recognizing it as a key strategy for achieving the United Nations Sustainable Development Goals (SDGs), particularly eradicating poverty (SDG 1), eliminating hunger (SDG 2), promoting decent work (SDG 8), and fostering social equality (SDG 10). Enhancing income levels improves farmers’ quality of life, reduces poverty, and ensures sustainable food production, thereby addressing hunger and malnutrition [1,2]. This is especially critical for smallholder and marginalized farmers, as income growth helps narrow disparities and reduce inequality. Furthermore, higher farm incomes contribute to rural economic development by making agricultural work more productive, stable, and capable of generating sustainable income. The Food and Agriculture Organization of the United Nations (FAO) asserts that agricultural trade enables food systems to use scarce natural resources, such as land and water, more efficiently and sustainably [3]. By facilitating the global dissemination of modern technologies, agricultural trade improves food security and nutrition. *The 2030 Agenda for Sustainable Development* recognizes international trade as a driver of inclusive economic growth and poverty reduction, emphasizing its central role in achieving the SDGs [4,5]. Thus, agricultural trade, as a key mechanism for integrating and leveraging domestic and international market resources, holds significant strategic and economic importance for developing countries and regions where agriculture remains the dominant sector.

However, amid unprecedented global changes, agricultural trade is facing several significant challenges. First, although most developing countries are primarily agricultural, the major agricultural exporters are developed countries, whereas developing nations remain the primary importers [6,7]. This results in a substantial imbalance in the distribution of global agricultural trade performance. Second, risks such as “decoupling” and disruptions in global agricultural supply chains have intensified, adding greater uncertainty to agricultural trade [8,9]. To address these challenges, many countries are leveraging the rapid development of digital technologies and the digital economy by investing in agricultural digitalization to unlock the potential of digital innovations in the sector. The FAO also recognizes the importance of digital transformation in the agricultural and food sectors. In 2022, the FAO joined the Digital Public Goods Alliance (DPGA) to promote digital public goods and outlined key areas for digital agriculture in its *2022–2031 Strategic Framework* [10]. As a new paradigm in agriculture, digitalization is expected to play a vital role in transforming traditional production methods, enhancing productivity, and increasing farmers’ incomes [11–13].

This study investigates how agricultural digitalization inputs influence the scale and incomes of agricultural exports, aiming to answer the following questions. First, do agricultural digitalization inputs from both domestic and foreign sources improve agricultural export performance, including gross exports (E), domestic value-added in exports (DVA), and the ratio of domestic value-added in exports to gross exports (EDVAR)? Second, are there differences in the mechanisms through which domestic and foreign digitalization inputs influence export performance? Third, does the impact of agricultural digitalization inputs differ between developing and developed economies?

## Literature review and theoretical framework

### Literature review

Research on export performance dates back to the seminal work by Tookey (1964) [14]. Over the past four decades, extensive literature has examined various dimensions of export performance. Specifically, agricultural export performance reflects how effectively a country or region sells agricultural products in global markets [15,16]. This concept serves as a broad indicator of success in agricultural trade, encompassing factors such as export volume and value, export growth rate, and the profitability and efficiency of export operations [17]. As global value chain (GVC) specialization deepens, intermediate goods undergo multiple rounds of cross-border production and trade. This complicates the use of traditional gross trade value-based accounting methods, which fail to reflect actual export performance accurately and may exaggerate imbalances in the distribution of trade benefits, thereby intensifying trade frictions [18,19]. To address this issue, some scholars have adopted the concept of “Value-Added in Trade” (VAiT) for statistical analysis [20,21]. As methods for measuring export performance continue to improve, scholars have reached a consensus on the key factors influencing agricultural export performance. According to cost–benefit theory, trade costs are undoubtedly a major factor influencing export performance. The deepening of GVC specialization, along with the expansion of intermediate goods trade, increases tariffs and transportation costs, thereby amplifying the effect of trade costs on export performance [22,23]. This amplification effect is more pronounced in emerging markets and less evident in most developed economies [24]. In addition, factors such as a country’s position in the GVC, land resources, human capital, and institutional constraints also influence agricultural export performance [25–27].

Early research on agricultural digitalization primarily focuses on its definition and effectiveness. Agricultural digitalization refers to the application of digital technologies and information tools—such as IoT, drones, remote sensing, big data, cloud computing, and artificial intelligence—to optimize various stages of agricultural production, management, and marketing [28,29]. It aims to enhance agricultural efficiency, improve product quality, and promote sustainable agricultural development. The benefits of agricultural digitalization include improving the quality and efficiency of the supply system, adapting more effectively to changing demand, and fostering a digitalized reproduction system [30]. These outcomes contribute to increased agricultural income and the advancement of agricultural modernization [31,32]. As the theoretical framework becomes more systematic and foundational data more comprehensive, the quantitative analysis of agricultural digitalization has emerged as a key research focus. One approach involves constructing a multidimensional indicator system and applying comprehensive evaluation methods to assess the level of agricultural digitalization development [33–35]. However, existing studies often use the provincial level as the unit of analysis. The second approach to measuring industrial digitalization focuses on digital investment, using the value-added or intermediate inputs from the information and communication industry to various sectors in the input–output table as indicators of digitalization [36–38]. However, existing research mainly focuses on the manufacturing sector. These methods of measuring digital investment provide useful references for evaluating agricultural digital investment in this study.

In recent years, the deep integration of the digital economy with agriculture has emerged as a significant trend in global agricultural innovation and development. Digital investment is regarded as a key factor in improving quality, reducing costs, and enhancing efficiency in agriculture [30]. Awareness of the empowering role of digital and information technologies in agriculture is growing, with digital empowerment increasingly recognized as a key driver of high-quality agricultural development [39,40]. Agricultural digital infrastructure helps eliminate information asymmetry, thereby improving the coordination of input–output structures and enhancing the integration of production organization systems [13]. Digital technologies, such as mobile communication and mobile payments, play a crucial role in enhancing market transparency for agricultural products, increasing output, and developing efficient logistics systems [41]. These technologies also enhance collaboration among stakeholders in agricultural water supply, thereby improving the efficiency and quality of irrigation systems [42,43]. However, the effects of agricultural digitalization inputs from domestic and foreign sources differ [44].

In summary, the existing literature affirms the critical role of agricultural digitalization in advancing agricultural modernization and high-quality development. However, few studies have examined the relationship between agricultural digitalization and export performance, and research differentiating between sources of digitalization inputs remains limited. This study utilizes the OECD Activity of Multinational Enterprises (AMNE) database and applies a trade value-added decomposition model [20] to analyze the export value-added of agricultural sectors across 76 economies. It employs three indicators—E, DVA, and EDVAR—to comprehensively describe agricultural export performance across economies. A two-way fixed effects model is then employed to assess the impact of agricultural digitalization inputs from different sources on export performance and to identify the underlying mechanisms.

This study makes the following key contributions. First, by recognizing the globalization and fragmentation of digital product production, it carefully examines the national origins of digital investments, distinguishing between domestic and foreign sources of agricultural digitalization. This distinction fills a gap in the existing literature, where digitalization is often treated as a homogeneous process, and enables a comprehensive and objective assessment of the effects of agricultural digitalization inputs, thereby enhancing the applicability and global relevance of the findings. Second, through a heterogeneity analysis of developed and developing economies, the study explores agricultural export performance across different stages of development, offering comparative insights that extend current understanding of how digital transformation shapes agricultural trade structures. The findings are particularly valuable for developing economies where agriculture remains a dominant sector. Third, this study employs three indicators—E, DVA, and EDVAR—to comprehensively measure agricultural export performance by integrating gross value and value-added approaches. By constructing a regression model to examine the effects of agricultural digitalization from different sources on these indicators, the study provides empirical evidence and policy implications for leveraging digitalization to promote global food security and sustainable development. Overall, by differentiating the sources of agricultural digitalization and exploring their heterogeneous impacts across economies, this study offers new insights into the mechanisms through which digital transformation enhances agricultural trade competitiveness.

### Theoretical framework

Agricultural digitalization refers to the integration of digital technologies across all stages of the agricultural value chain, transforming production tools and methods to enhance overall efficiency [29]. Key digital innovations—such as sensors, satellites, robots, and drones—have significantly reshaped agriculture and its related value chains [28]. Agricultural digitalization is increasingly recognized as a crucial production input in the agricultural GVC, originating from both domestic and foreign sources. The national origin of these inputs influences economic agents’ capacity to lead and manage international labor divisions, thus affecting factor income distribution [38]. Consequently, the impact of agricultural digitalization inputs on export performance may vary depending on investment origin.

The direct contribution of agricultural digitalization to agricultural export performance is primarily reflected in five aspects. First, it contributes to strengthening economies of scale. In traditional agricultural production and operations, economies of scale primarily depend on expanding the scale of land management. However, small farmers face challenges such as rising supervisory costs, increased natural risks, and greater market uncertainties when attempting to scale up [45]. As the marginal cost of adding users to digital systems is minimal, and with the continued penetration of digital technologies in agriculture, the sector has begun to exhibit characteristics of high fixed costs and low marginal costs [46]. Small farmers increasingly have access to scalable digital services, enabling them to benefit from economies of scale without expanding their land holdings [47]. This suggests that agricultural digitalization lowers the barriers for small farmers to participate in economies of scale while also diversifying the forms of large-scale agricultural operations. Second, agricultural digitalization contributes to achieving economies of scope. On the one hand, digital technologies enable producers to leverage the cyclical and seasonal nature of agricultural production to develop joint production plans, establish shared brands, and prevent underutilization of resources [48]. On the other hand, continuous extraction and application of agricultural data have supported the expansion of multi-variety, small-batch production models and precision marketing strategies based on consumer profiles [49,50]. This strengthens the link between the supply and demand sides of agricultural products, thereby facilitating the achievement of economies of scope. Third, agricultural digitalization helps reduce information asymmetry. According to information economics, information symmetry facilitates better production and pricing decisions by firms. Big data technologies integrate weather data, crop health data, and global market demand to deliver accurate yield forecasts and market analyses [51]. This enables producers to adjust production plans, optimize export strategies, and maximize revenues. Blockchain and IoT technologies enhance transparency in the agricultural production process, enabling traceability from farm to table [52]. This helps overcome export barriers, such as those imposed by the Sanitary and Phytosanitary Measures(SPS) Agreement, thereby increasing agricultural exports [53]. Additionally, big data platforms collect real-time information on global agricultural price fluctuations, which, when combined with logistics and international demand data, enable exporters to make informed pricing decisions and avoid losses due to excessively low or high prices [54]. Fourth, agricultural digitalization enhances the efficiency of supply chains and logistics. IoT monitoring, sensor technologies, and data tracking support smart warehousing and logistics management [55,56]. These technologies monitor humidity and temperature during storage and transportation, reducing the risk of spoilage or damage and ensuring product freshness. Efficient logistics and supply chain systems facilitate rapid delivery of agricultural products to target markets, minimize the risk of export returns due to quality issues, and safeguard export revenues [57,58]. Fifth, agricultural digitalization contributes to increasing product added value. Blockchain technology records production and logistics data for each batch of agricultural products, including planting times, pesticides used, and storage and transportation conditions [59–61]. This information is accessible to exporters and consumers, reducing uncertainty about product quality and fostering trust. The transparency and traceability provided enhance the market value of agricultural products, especially in high-end markets in developed countries where consumers are more willing to pay a premium for products with reliable production records and green certification. Thus, we propose the following hypotheses:

***Hypothesis 1:*** *Agricultural digitalization inputs enhance agricultural export performance*

Domestic agricultural digitalization inputs primarily focus on improving infrastructure and promoting widespread technology adoption. On the one hand, government-led investments establish essential information and communication infrastructure in rural areas, such as broadband networks and smart agricultural machinery [32,62]. These investments lay the foundation for agricultural digital transformation by optimizing planting, irrigation, and harvesting, thereby improving productivity. On the other hand, policy incentives and subsidies encourage farmers and agricultural enterprises to adopt digital technologies [63]. This reduces barriers to digital technology adoption and enhances production and supply chain management through integrated agricultural data platforms, ensuring a stable supply of high-quality agricultural exports [53]. In summary, domestic agricultural digitalization inputs target production-side improvements to enhance agricultural efficiency. According to new-new trade theory, as total factor productivity increases, firms surpass the export productivity threshold, enabling more agricultural enterprises to export and thus increase export revenues [64]. Accordingly, we propose the following hypotheses:

***Hypothesis 2*** *Domestic agricultural digitalization inputs enhance agricultural export performance by increasing production efficiency.*

The existing multilateral trade framework exhibits inconsistencies in governing digital trade barriers, particularly those related to digital infrastructure connectivity [46]. As a result, the share of foreign digital factor inputs is often insufficient to significantly influence domestic productivity. Moreover, foreign digitalization inputs and domestic production factors require a period of adjustment, leading to no immediate improvement in production efficiency [65]. However, foreign agricultural digitalization inputs typically aim to enhance supply chain efficiency, particularly through the use of big data and blockchain technologies to track agricultural production, transportation, and sales, thereby improving transparency and efficiency [52]. Optimizing supply chain processes reduces transportation and storage costs in agricultural trade, shortens the time for products to reach international markets, and directly enhances export competitiveness [31]. Furthermore, foreign agricultural digitalization inputs facilitate access to valuable information on consumer preferences, demand, and market trends, thereby reducing information costs and trade uncertainty. Therefore, foreign agricultural digitalization inputs primarily target the consumption side, focusing on optimizing supply chains and addressing market demand to reduce trade costs and enhance competitiveness [8]. Accordingly, we propose the following hypotheses:

***Hypothesis 3*** *Foreign agricultural digitalization inputs enhance agricultural export performance through the reduction of trade costs.*

## Research design

### Variable selection and measurement

#### Explained variables.

To provide a comprehensive evaluation, this study employs three statistical indicators to measure agricultural export performance: DVA, E, and EDVAR. The measurement methods follow the gross trade accounting approach proposed by Koopman et al. (2014) [20]and decompose the gross exports of domestic agricultural enterprises across 76 economies from the OECD AMNE database. DVA represents the domestic value-added in agricultural exports absorbed by foreign markets, essentially reflecting the “real” value exchanged in trade generated by a country’s domestic production factors involved in the GVC. E refers to the gross export value as captured by traditional trade statistics. EDVAR is the export domestic value-added ratio, which indicates the proportion of domestic value-added in gross exports. This ratio reflects a country’s ability to create value within its exports and, to some extent, its international competitiveness.

#### Explanatory variables.

According to the U.S. Bureau of Economic Analysis (BEA), the digital economy comprises three components: (1) digital-enabling infrastructure, (2) digital transactions, and (3) digital media [38]. Industrial digitalization refers to integrating digital technologies into traditional sectors to improve efficiency and create value. As e-commerce is not separately classified in the International Standard Industrial Classification (ISIC Rev. 4.0)—being mainly embedded in G47 (retail trade) and J61–J63 (information and communication) sectors—this study focuses on two functional dimensions: digital infrastructure and digital media. As shown in Table 1, digital infrastructure comprises C26 and J61–J63, which provide the technological foundation for agricultural digital transformation. Digital media includes J58–J60, which support agricultural knowledge diffusion and digital marketing. This classification aligns with the OECD digital economy taxonomy and captures both the technological and informational dimensions of digitalization. The level of domestic agricultural digitalization inputs is defined as the ratio of the digitalization inputs consumption coefficient in the agricultural sector to the total consumption coefficient across all sectors, calculated using OECD AMNE data. This can be expressed as follows:

**Table 1:** Classification of digitalization inputs.

Classification	Content	Industry Codes and Description (ISIC Rev. 4.0)
Digital Infrastructure	Telecommunications Equipment and Services	J61: Telecommunications; J62: Computer programming, consultancy and related activities; J63: Information service activities.
	Computer Software	
	Computer Hardware	C26: Manufacture of computer, electronic and optical products.
Digital Media	Publishing	J58: Publishing activities; J59: Motion picture, video and television programme production, sound recording and music publishing activities; J60: Programming and broadcasting activities.
	Internet and Broadcasting	
	Traffic and Downloads	


$${dg}^{r}=\frac{\sum_{i}^{T}b_{ij}^{rr}}{\sum_{s}^{N}\sum_{i}^{M}b_{ij}^{sr}}$$ (1)

In equation (1), ${dg}^{r}$ represents the level of domestic agricultural digitalization inputs in country *r*; *j* denotes the agricultural sector; *T* refers to the digitalization sectors in the input-output table (C26, J58–J63); $\sum_{i}^{T}b_{ij}^{rr}$ is the sum of the complete consumption coefficients for domestic digitalization inputs into the agricultural sector of country *r*; *M* represents all sectors in the input-output table; *N* refers to all countries; and $\sum_{s}^{N}\sum_{i}^{M}b_{ij}^{sr}$ denotes the sum of the complete consumption coefficients for digital inputs from all sectors and countries into the agricultural sector of country *r*.

Similarly, the level of foreign agricultural digitalization inputs is measured by the ratio of the complete consumption coefficient of foreign digitalization inputs into the domestic agricultural sector to the total consumption coefficient of all factors. The corresponding expression is as follows:


$$\begin{array}{c} {fg}^{r}=\frac{\sum_{s\neq r}^{N}\sum_{i}^{T}b_{ij}^{sr}}{\sum_{s}^{N}\sum_{i}^{M}b_{ij}^{sr}} \end{array}$$ (2)

In Equation (2), ${fg}^{r}$ represents the level of foreign agricultural digitalization inputs in country *r*, and $\sum_{s\neq r}^{N}\sum_{i}^{T}b_{ij}^{sr}$ represents the sum of the complete consumption coefficients of foreign digitalization inputs into the agricultural sector of country *r.*

#### Control variables.

To mitigate the impact of omitted variables on model estimation, the following control variables are included: Agricultural GVC position (*GP*), defined as the ratio of a country’s forward to backward agricultural participation in the GVC. Technological level (*te*) is defined as the proportion of medium- and high-tech exports to total manufactured exports, sourced from the World Bank’s World Development Indicators (WDI) database. This measure reflects a country’s technological sophistication and innovation capacity. Institutional quality (*rq*) is assessed using the Regulatory Quality index from the Worldwide Governance Indicators (WGI) database. This index captures perceptions of the government’s ability to design and implement effective policies and regulations. Human capital (*hc*), which plays a critical role in production configuration, technological innovation, and technology absorption, is essential for adapting to digital transformation and exhibits strong knowledge and technological spillover effects, thereby enhancing agricultural export performance. Human capital is quantified using the human capital index from the Penn World Table (version 10.01). Land resources (*lr*) are measured by the share of arable land, obtained from the World Bank database.

#### Mechanism variables.

Agricultural production efficiency (*pe*) is used to represent the productivity effect and to examine the mechanism through which domestic agricultural digitalization inputs affect agricultural export performance. The agricultural production index (2014–2016 = 100), adjusted for constant prices in the FAO database, reflects real changes in agricultural output. Accordingly, this study uses this indicator to measure agricultural production efficiency.

Agricultural trade costs (*tc*) are used to represent the trade cost effect and to examine the mechanism through which foreign agricultural digitalization inputs affect agricultural export performance. Bilateral trade cost data for over 180 countries from 1995 to 2021 are provided by the Economic and Social Commission for Asia and the Pacific (ESCAP), in collaboration with the World Bank. The bilateral trade costs in this database are calculated using the Novy (2013) [66] model, as shown in Equation (3).


$$T_{ij}={(\frac{x_{ii}x_{jj}}{x_{ij}x_{ji}})}^{\frac{t}{\text{2}(\;\sigma -\text{1})}}-\text{1}$$ (3)

In Equation (3), $x_{ii}$ and $x_{jj}$ represent the domestic trade of countries *i* and *j*, respectively; $x_{ij}$ denotes the exports of country *i* to country *j*; $T_{ij}$ indicates the bilateral trade cost between countries *i* and *j*; and σ refers to the elasticity of substitution for goods, which is greater than 1.

This study further uses export data from the OECD AMNE database to calculate the weighted average trade cost of a country’s agricultural exports. The calculation uses the share of agricultural exports to each destination country, relative to total agricultural exports, as the weight, as shown in Equation (4).


$${TC}_{i}\;=\sum_{j}\frac{x_{ij}}{x_{i}}T_{ij}$$ (4)

In Equation (4), $x_{i}$ denotes the total agricultural exports of country *i*; $x_{ij}$ represents the agricultural exports from country *i* to country *j*; and ${TC}_{i}$ refers to the weighted average trade cost of country *i*’s agricultural exports. Since the calculated trade cost values are relatively large, their logarithmic values are used in this study.

### Econometric model

This study employs a panel model to analyze the data structure, characterized by a large cross-sectional dimension (N) and a small time dimension (T). First, a unit root test is conducted on the panel data prior to the baseline regression. The results strongly reject the null hypothesis, indicating the absence of a unit root. Second, the Least Squares Dummy Variable (LSDV) method is applied, revealing that most individual dummy variables are significant, indicating the presence of individual effects. Therefore, the mixed-effects regression model is deemed inappropriate. To choose between the fixed-effects and random-effects models, a Hausman test is conducted, yielding a p-value of 0, which strongly rejects the random-effects model. Consequently, the fixed-effects model is adopted. Additionally, the differencing method reveals the presence of autocorrelation in the panel data. The time effect test strongly rejects the null hypothesis of no time effect, leading to the inclusion of time effects in the model. Finally, the White test indicates heteroscedasticity; therefore, a two-way fixed-effects model with cluster-robust standard errors at the country level is employed. The model is as follows.


$$Y_{it}=a_{\text{0}}+a_{\text{1}}{dg}_{it}+a_{\text{2}}{fg}_{it}+a_{\text{3}}{Control}_{it}+r_{i}+\lambda_{t}+\varepsilon_{it}\;\;$$ (5)

In Equation (5), the subscript *i* denotes the country, and *t* denotes the year. *Y* represents the dependent variables, including *DVA*, *E*, and *EDVAR*. *dg* refers to domestic agricultural digitalization inputs; *fg* refers to foreign agricultural digitalization inputs; and *Control* represents the control variables, which include the position in the agricultural GVC (*GP*), technological level (*te*), institutional quality (*rq*), human capital (*hc*), and land resources (*lr*). $r_{i}$ denotes individual fixed effects, $\lambda_{t}$ denotes time fixed effects, and $\varepsilon_{it}$ denotes the random error term. To mitigate the adverse effects of inconsistent variable units and heteroscedasticity, natural logarithms are applied to domestic value-added in exports, gross exports, and human capital, expressed as ln (*DVA*), ln (*E*), and ln (*hc*).

### Data sources and descriptive statistics

The OECD AMNE database, released in 2023, contains input–output data for 76 economies. However, due to missing data for certain variables in Belarus and the Taiwan region of China, the regression analysis is conducted using the remaining 74 economies (The list of 74 economies is provided in Table A in S2 Appendix). These include major developed and developing economies worldwide. The study covers the period from 2000 to 2019, comprising 20 years of data. The sample consists of 1,480 observations, and descriptive statistics for the relevant variables are reported in Table 2. The table is organized by variable type, including dependent, explanatory, mediating, and control variables, as defined in the “variable selection and measurement” section. Each variable’s mean, standard deviation, minimum, and maximum values are reported to summarize its distribution and variation across economies and over time. Additionally, a correlation matrix is used to examine the models, and the variance inflation factor (VIF) is employed to test for multicollinearity. The correlation coefficients between variables are generally below 0.6, and the VIF values are significantly lower than 5, indicating that multicollinearity is not a concern. Due to space limitations, the results of the multicollinearity test are provided in Table B in S2 Appendix.

**Table 2:** Descriptive statistics of the variables.

Variables	Mean	Std	Max	Min
domestic value-added in exports (*DVA*)	2998.15	5582.04	50169.96	0.13
gross exports (*E*)	3561.39	6487.17	57866.92	0.18
the ratio of domestic value-added in exports to gross exports (*EDVAR*)	0.82	0.10	1.00	0.41
domestic agricultural digitalization inputs (*dg*)	0.01	0.01	0.09	0.00
foreign agricultural digitalization inputs (*fg*)	0.02	0.01	0.05	0.00
agricultural production efficiency (*pe*)	0.91	0.15	1.92	0.35
agricultural trade costs (*tc*)	152.13	52.74	499.80	37.73
agricultural GVC position (*GP*)	1.22	0.89	12.83	0.18
technological level (*te*)	0.45	0.21	0.87	0.00
institutional quality (*rq*)	0.68	0.25	1.00	0.01
human capital (*hc*)	2.84	0.62	4.35	1.31
land resources (*lr*)	0.18	0.15	0.64	0.00

## Empirical result

### Benchmark regression result

The main factors influencing agricultural export performance are analyzed using a two-way fixed-effects model, and the baseline regression results are presented in Table 3. The results indicate that domestic agricultural digitalization inputs exert a significant positive impact on both DVA and E, with coefficients of 11.09 and 10.19, respectively. It also exerts a significant positive effect on EDVAR. These findings suggest that increasing domestic agricultural digitalization inputs foster the growth of both total agricultural exports and domestic value-added absorbed by foreign markets. Moreover, the domestic value-added component grows at a faster rate, thereby enhancing a country’s international agricultural competitiveness. Foreign agricultural digitalization inputs exert a positive effect on both DVA and E but a negative effect on EDVAR. This indicates that although foreign agricultural digitalization inputs promote the growth of total agricultural exports and domestic value-added, the foreign value-added component increases at a faster rate and captures a larger share of the resulting profits. Although domestic and foreign agricultural digitalization inputs have different effects on agricultural export performance, both contribute to its overall growth, thereby confirming Hypothesis 1.

**Table 3:** Benchmark regression results.

Variables	(1)	(2)	(3)	(4)	(5)	(6)
	ln (*DVA*)	ln (*DVA*)	ln (*E*)	ln (*E*)	*EDVAR*	*EDVAR*
*dg*	${\text{12}.\text{9317}}^{**}$ (2.17)	${\text{11}.\text{0904}}^{**}$ (2.44)	${\text{12}.\text{0953}}^{**}$ (2.04)	${\text{10}.\text{1894}}^{**}$ (2.23)	${\text{0}.\text{7689}}^{**}$ (2.33)	${\text{0}.\text{8292}}^{**}$ (2.56)
*fg*	8.0996 (0.78)	${\text{15}.\text{5869}}^{*}$ (1.70)	14.4901 (1.37)	${\text{21}.\text{6840}}^{**}$ (2.31)	${-\text{4}.\text{7090}}^{**}$ (−6.44)	${-\text{4}.\text{4416}}^{***}$ (−6.06)
*GP*		${\text{0}.\text{2354}}^{**}$ (2.53)		${\text{0}.\text{2261}}^{**}$ (2.62)		0.0088 (1.31)
*te*		0.2815 (0.81)		0.2847 (0.80)		−0.0088 (−0.43)
*rq*		${\text{1}.\text{0093}}^{**}$ (2.34)		${\text{0}.\text{9804}}^{**}$ (2.25)		0.0213 (0.89)
ln (*hc*)		${\text{1}.\text{3520}}^{**}$ (2.08)		${\text{1}.\text{3598}}^{*}$ (1.96)		0.0083 (0.12)
*lr*		${\text{2}.\text{8155}}^{**}$ (2.28)		${\text{2}.\text{8789}}^{**}$ (2.34)		0.0017 (0.01)
Constant Term	${\text{5}.\text{8057}}^{***}$ (48.11)	${\text{2}.\text{7254}}^{***}$ (3.35)	${\text{5}.\text{9728}}^{***}$ (48.66)	${\text{2}.\text{8278}}^{***}$ (3.24)	${\text{0}.\text{8475}}^{***}$ (83.89)	${\text{0}.\text{8720}}^{***}$ (10.00)
Individual Fixed Effects	Fixed	Fixed	Fixed	Fixed	Fixed	Fixed
Time Fixed Effects	Fixed	Fixed	Fixed	Fixed	Fixed	Fixed
Sample Size	1480	1404	1480	1404	1480	1404
${\text{R}}^{\text{2}}$	0.64	0.67	0.66	0.68	0.25	0.43

***; **; * indicate significance at the 1%, 5%, and 10% confidence levels, respectively, with the t-statistics shown in parentheses. The same applies to the following text.

The results for the control variables indicate that the position in the agricultural GVC has a significant positive effect on both DVA and E, and also exerts a positive influence on EDVAR. This suggests that a higher position in the agricultural GVC enhances a country’s capacity to generate domestic value-added in agriculture. Institutional quality, human capital, and land resources all have positive effects on DVA and E, and also exert positive influences on EDVAR. These findings suggest that fostering a favorable agricultural business environment, cultivating agricultural talent, and effectively managing land resources can significantly enhance a country’s agricultural export performance. Although the effect of technological level on DVA and E is positive, it is not statistically significant.

### Heterogeneity examination

The development of agricultural digitalization worldwide is uneven, following diverse paths shaped by resource endowments, technological foundations, farm characteristics, and farmer preferences, resulting in varying levels, forms, and approaches. Agricultural digitalization follows two distinct models in developed and developing countries. In developed countries and emerging economies, technological adoption in agriculture is already highly advanced. For example, precision agriculture, an innovation-driven model, is gaining traction in many developed countries, where large farms capitalize on technological applications to achieve economies of scale and higher returns on investment [67]. However, the adoption of digital technologies remains limited in some developing countries. In many cases, agricultural technology use in these countries is limited to sending mobile phone text messages or playing offline digital videos to deliver information to rural farmers [68]. Therefore, this study classifies the 74 countries and regions into developed and developing economies to further examine the impact of agricultural digitalization inputs on agricultural export performance across different economic contexts.

Regression results for developing and developed economies are presented in Table 4. Results indicate that domestic agricultural digitalization inputs have a significantly positive effect on agricultural export performance in developing economies, with a stronger impact than in developed economies. This suggests that developing economies can more effectively enhance their agricultural export competitiveness and promote agricultural export performance by increasing domestic agricultural digitalization inputs. Foreign agricultural digitalization inputs have a significant positive effect on agricultural DVA and E in both developing and developed economies, but a negative effect on EDVAR in both. This indicates that although foreign agricultural digitalization inputs promote export growth, the foreign value-added component grows more rapidly. This finding aligns with the baseline regression results.

**Table 4:** Heterogeneity analysis across economies.

Variables	Developing Economies			Developed Economies		
	(1)	(2)	(3)	(4)	(5)	(6)
	ln (*DVA*)	ln (*E*)	*EDVAR*	ln (*DVA*)	ln (*E*)	*EDVAR*
*dg*	${\text{11}.\text{9173}}^{**}$ (2.09)	${\text{10}.\text{6462}}^{*}$ (1.82)	${\text{1}.\text{0129}}^{***}$ (3.07)	1.4804 (0.28)	1.6026 (0.28)	0.2523 (0.42)
*fg*	${\text{31}.\text{7808}}^{**}$ (2.25)	${\text{36}.\text{8926}}^{**}$ (2.48)	${-\text{4}.\text{3008}}^{***}$ (−4.64)	${\text{25}.\text{8088}}^{**}$ (2.13)	${\text{31}.\text{7040}}^{***}$ (2.73)	${-\text{3}.\text{7301}}^{***}$ (−3.91)
*GP*	${\text{0}.\text{2114}}^{***}$ (3.03)	${\text{0}.\text{2047}}^{***}$ (3.11)	0.0062 (1.30)	${\text{1}.\text{1581}}^{***}$ (5.23)	${\text{1}.\text{0757}}^{***}$ (4.97)	${\text{1}.\text{0623}}^{***}$ (3.95)
*te*	0.2845 (0.64)	0.3368 (0.73)	${-\text{0}.\text{0420}}^{**}$ (−2.08)	−0.3663 (−0.70)	−0.4242 (−0.79)	0.0371 (1.05)
*rq*	0.6552 (1.47)	0.6727 (1.45)	−0.0102 (−0.38)	${\text{1}.\text{0810}}^{*}$ (0.93)	1.0460 (1.61)	0.0170 (0.31)
ln *(hc)*	0.9786 (0.95)	1.0943 (1.00)	−0.0860 (−1.06)	0.2662 (0.44)	0.2659 (0.51)	0.0275 (0.27)
*lr*	1.4671 (0.79)	1.3628 (0.73)	0.1195 (0.70)	${\text{2}.\text{7211}}^{*}$ (1.70)	${\text{2}.\text{9861}}^{*}$ (1.85)	−0.1459 (−1.19)
Constant Term	${\text{3}.\text{7327}}^{***}$ (3.30)	${\text{3}.\text{7156}}^{***}$ (3.08)	${\text{0}.\text{9872}}^{***}$ (10.93)	${\text{2}.\text{8095}}^{***}$ (3.15)	${\text{3}.\text{0264}}^{***}$ (3.54)	${\text{0}.\text{7580}}^{***}$ (5.75)
Individual Fixed Effects	Fixed	Fixed	Fixed	Fixed	Fixed	Fixed
Time Fixed Effects	Fixed	Fixed	Fixed	Fixed	Fixed	Fixed
Sample Size	720	720	720	684	684	684
${\text{R}}^{\text{2}}$	0.76	0.76	0.41	0.66	0.67	0.55

Results for control variables show that the position in the agricultural GVC has a significant positive effect on agricultural export performance in both developing and developed economies. This effect is more pronounced in developed economies, suggesting that by leveraging advanced technologies and market information, these economies are highly integrated into the agricultural GVC and can capture greater value-added trade benefits. Institutional quality and land resources have a positive effect on DVA and E in developed economies, with a stronger impact than in developing economies. Effective institutional frameworks enhance digital technology accessibility, thereby driving improvements in agricultural productivity and income growth. The World Bank’s “Empowering Agriculture” project compares institutional regulatory requirements for information and communication technology across countries, highlighting that most low-income nations lack relevant standards, while high-income OECD countries have implemented robust frameworks encouraging the private sector to strengthen connectivity beyond urban centers. Furthermore, developed economies leverage their industrial capacity to utilize land resources more efficiently, driving income growth.

### Endogeneity analysis

It is assumed that improved agricultural export performance may incentivize governments and farmers to reinvest income into agricultural digitalization, implying potential bidirectional causality between export performance and digitalization inputs. To address potential endogeneity concerns and ensure the reliability of the empirical results, this study uses the following instrumental variables. First, the interaction term between the one-period lag of domestic agricultural digitalization inputs and the internet penetration rate (IV1) is used as the instrumental variable for domestic agricultural digitalization inputs. Agricultural digitalization inputs show a certain degree of persistence, as past input levels may affect current input decisions but are unlikely to directly influence current agricultural export performance. In addition, agricultural digitalization inputs strongly depend on internet infrastructure, while the expansion of internet penetration is usually driven by top-down national policies that are largely exogenous. Therefore, IV1 is strongly correlated with agricultural digitalization inputs and satisfies the exclusion restriction, confirming it as a valid instrumental variable. Second, this study constructs a weighted sum of the inverse geographical distances between national capitals and the corresponding countries’ domestic agricultural digitalization inputs, which is used as the instrumental variable for foreign agricultural digitalization inputs (IV2). The geographical distance between national capitals is a strictly exogenous variable that meets the exclusion condition. Moreover, when geographically closer countries have higher levels of digitalization inputs, a country is more likely to import digital products from these countries. Hence, IV2 is expected to be strongly correlated with foreign agricultural digitalization inputs. Furthermore, this study uses the weighted sum of the inverse geographical distances between national capitals and the corresponding countries’ total trade as a share of GDP as another instrumental variable for foreign agricultural digitalization inputs (IV3). Geographically proximate countries tend to engage in more active trade, and a higher level of bilateral trade increases the likelihood that a country will import digital products from its neighbors, thereby influencing its level of agricultural digitalization inputs.

The specific calculation formulas for IV2 and IV3 are presented in Equations (6) and (7).


$${IV\text{2}}_{it}=\sum_{j}^{n}(\frac{\text{1}}{{distance}_{ij}}\times {dg}_{jt})$$ (6)

$${IV\text{3}}_{it}=\sum_{j}^{n}(\frac{\text{1}}{{distance}_{ij}}\times {EM}_{jt})$$ (7)

Among them, $IV{\text{2}}_{it}\;$and $IV{\text{3}}_{it}\;$represent the instrumental variables for foreign agricultural digitalization inputs of economy i in period t; $distance_{ij}\;$denotes the geographical distance between the capitals of economies i and j; $dg_{jt}\;$refers to the domestic agricultural digitalization inputs of economy j in period t; and $EM_{jt}$ indicates the share of total goods and services trade in GDP for economy j in period t. Data on internet penetration and the ratio of goods and services trade to GDP are obtained from the World Bank database, while data on distances between national capitals are derived from the CPEII database.

Based on the above discussion, endogeneity tests were conducted, and the results are presented in Table 5 and Table 6. First, in the under-identification test, the Kleibergen–Paap rk LM statistic (KP LM) passed the test at the 1% significance level, indicating that the selected instrumental variables do not suffer from identification deficiencies. Second, in the weak instrumental variable test, the Kleibergen–Paap rk Wald F statistic (KP F) exceeded the critical value corresponding to the 10% significance level, suggesting that the two types of instrumental variables constructed in this study are not subject to weak identification. Furthermore, the first-stage regression results show that the estimated coefficients of all instrumental variables are positive and significant at the 1% level, demonstrating their strong correlation with the endogenous explanatory variables. In the second-stage regression results, the coefficients of domestic and foreign agricultural digitalization inputs have consistent signs and similar levels of significance, further confirming the robustness of the model estimation results.

**Table 5:** Results of endogeneity test (I).

Variables	IV(2SLS)				
	1^st^-stage		2^nd^-stage		
	*dg*	*fg*	ln(*DVA*)	ln(*E*)	*EDVAR*
*dg*			${\text{10}.\text{3255}}^{**}$ (2.00)	${\text{10}.\text{3752}}^{*}$ (1.92)	0.1484 (0.26)
*IV1*	${\text{0}.\text{7533}}^{***}$ (8.91)				
*fg*			${\text{48}.\text{8879}}^{***}$ (3.10)	${\text{62}.\text{4308}}^{***}$ (3.99)	${-\text{9}.\text{0695}}^{***}$ (−8.25)
*IV2*		${\text{0}.\text{5472}}^{***}$ (9.54)			
Controls	Yes	Yes	Yes	Yes	Yes
Individual Fixed Effects	Fixed	Fixed	Fixed	Fixed	Fixed
Time Fixed Effects	Fixed	Fixed	Fixed	Fixed	Fixed
F test	${\text{5}\text{5}.\text{8}\text{7}}^{***}$	${\text{4}\text{6}.\text{5}\text{7}}^{***}$	${\text{7}\text{2}.\text{2}\text{1}}^{***}$	${\text{7}\text{5}.\text{2}\text{0}}^{***}$	${\text{1}\text{8}.\text{4}\text{1}}^{***}$
KP LM	${\text{6}\text{4}.\text{8}\text{5}}^{***}$				
KP F	34.07 (7.03)				
Sample Size	1326	1326	1326	1326	1326
${\text{R}}^{\text{2}}$			0.61	0.60	0.17

**Table 6:** Results of Endogeneity Test (II).

Variables	IV(2SLS)				
	1^st^-stage		2^nd^-stage		
	*dg*	*fg*	ln(*DVA*)	ln(*E*)	*EDVAR*
*dg*			${\text{10}.\text{0263}}^{*}$ (1.94)	${\text{9}.\text{8224}}^{*}$ (1.77)	0.3291 (0.48)
*IV1*	${\text{0}.\text{7479}}^{***}$ (8.87)				
*fg*			${\text{52}.\text{3801}}^{***}$ (2.80)	${\text{68}.\text{8816}}^{***}$ (3.64)	${-\text{11}.\text{1789}}^{***}$ (−8.17)
*IV3*		${\text{0}.\text{3052}}^{***}$ (8.11)			
Controls	Yes	Yes	Yes	Yes	Yes
Individual Fixed Effects	Fixed	Fixed	Fixed	Fixed	Fixed
Time Fixed Effects	Fixed	Fixed	Fixed	Fixed	Fixed
F test	${\text{6}\text{5}.\text{5}\text{6}}^{***}$	${\text{3}\text{3}.\text{3}\text{4}}^{***}$	${\text{7}\text{5}.\text{0}\text{2}}^{***}$	${\text{7}\text{7}.\text{5}\text{6}}^{***}$	${\text{1}\text{7}.\text{9}\text{5}}^{***}$
KP LM	${\text{4}\text{7}.\text{3}\text{9}}^{***}$				
KP F	23.33 (7.03)				
Sample Size	1326	1326	1326	1326	1326
${\text{R}}^{\text{2}}$			0.60	0.59	0.06

### Robustness tests

To further verify the reliability of the baseline regression results, this study performs robustness checks. The results are shown in Table 7.

**Table 7:** Results of the robustness tests.

	Substitution of Dependent Variables		Substitution of Independent Variables		Winsorization Treatment		Multidimensional Clustering	
	(1)	(2)	(3)	(4)	(5)	(6)	(7)	(8)
	ln(*DVA*)	ln(*E*)	ln(*DVA*)	ln(*E*)	ln(*DVA*)	ln(*E*)	ln(*DVA*)	ln(*E*)
*dg*	${\text{16}.\text{5108}}^{***}$ (2.76)	${\text{16}.\text{2019}}^{***}$ (2.75)	${\text{1}.\text{6314}}^{**}$ (2.59)	${\text{1}.\text{7138}}^{***}$ (2.67)	${\text{12}.\text{0452}}^{**}$ (1.98)	${\text{11}.\text{4152}}^{*}$ (1.88)	${\text{11}.\text{0904}}^{**}$ (2.28)	${\text{10}.\text{1894}}^{*}$ (2.09)
*fg*	7.8080 (0.73)	14.0811 (1.26)	${\text{19}.\text{9614}}^{**}$ (2.07)	${\text{25}.\text{8513}}^{***}$ (2.66)	${\text{18}.\text{0972}}^{*}$ (1.77)	${\text{24}.\text{5600}}^{**}$ (2.32)	11.5869 (1.68)	${\text{21}.\text{6840}}^{**}$ (2.26)
Controls	Yes	Yes	Yes	Yes	Yes	Yes	Yes	Yes
Individual Fixed Effects	Fixed	Fixed	Fixed	Fixed	Fixed	Fixed	Fixed	Fixed
Time Fixed Effects	Fixed	Fixed	Fixed	Fixed	Fixed	Fixed	Fixed	Fixed
Sample Size	1168	1168	1404	1404	1404	1404	1404	1404
${\text{R}}^{\text{2}}$	0.72	0.73	0.67	0.69	0.67	0.68	0.14	0.14

**Note:** Due to space constraints, the regression results for the dependent variable EDVAR and the outcomes of the sequential exclusion test are provided in Table C and Table D in S2 Appendix, respectively.

First, the dependent variable is replaced with national agricultural export data from the UIBE GVC database, constructed by the University of International Business and Economics (UIBE) using the OECD input-output table. The regression results appear in columns (1) and (2) of Table 7. Second, the explanatory variable is substituted. Domestic agricultural digitalization inputs primarily emphasize digital infrastructure development, closely linked to national communication infrastructure. Thus, the Information and Communication Technology (ICT) Development Index from the UNCTAD statistical database is used as a proxy for domestic agricultural digitalization inputs in the regression analysis. Results are presented in columns (3) and (4) of Table 7. Third, winsorization is applied to mitigate outlier effects. Both explanatory and dependent variables are winsorized at the top and bottom 1%, with corresponding regression results shown in columns (5) and (6) of Table 7. Fourth, a multidimensional clustering approach involving both individual and time dimensions is applied, and the regression results are presented in columns (7) and (8) of Table 7. Fifth, a sequential exclusion approach for high-leverage countries is adopted. Specifically, the top ten countries in terms of agricultural domestic sector export value from 2000 to 2019 (USA, BRA, CAN, FRA, NLD, ESP, CHN, AUS, RUS, and ARG) are excluded one by one, and the regressions are re-estimated to evaluate the sensitivity of the model’s conclusions to changes in sample composition. The results show that, regardless of which country is excluded, the signs and significance levels of the coefficients for both domestic and foreign agricultural digitalization inputs remain consistent with those of the baseline regression, further confirming the robustness and generalizability of the findings. The results in Table 7 indicate that the signs and significance of coefficients for domestic and foreign agricultural digitalization inputs on agricultural export performance remain consistent with the baseline regression, confirming robustness.

### Mechanism tests

To validate Hypotheses 2 and 3, this study adopts the mediation effect framework proposed by Zhang et al. (2009) [69]. Agricultural production efficiency and trade costs are used as mediating variables in the following mediation model.


$$M_{it}=\theta_{\text{0}}+\theta_{\text{1}}{dg}_{it}+\theta_{\text{2}}{fg}_{it}+\theta_{\text{3}}{Control}_{it}+r_{i}+\lambda_{t}+\varepsilon_{it}$$ (8)

$$Y_{it}=a_{\text{0}}+a_{\text{1}}{dg}_{it}+a_{\text{2}}{fg}_{it}+a_{\text{3}}{Control}_{it}+a_{\text{4}}M_{it}+r_{i}+\lambda_{t}+\varepsilon_{it}$$ (9)

In Equations (8) and (9), *M* represents the mediating variables, namely agricultural production efficiency (*pe*) and agricultural trade costs (*tc*). The definitions of the remaining variables are consistent with those previously defined.

Table 8 presents the regression results of the mediation effect model for agricultural production efficiency.

**Table 8:** Mediation regression results for agricultural production efficiency.

Variables	(1)	(2)	(3)	(4)
	*pe*	*ln (DVA)*	*ln (E)*	*EDVAR*
*pe*		$\text{0}{.\text{4543}}^{*}$ (1.73)	0.3939 (1.45)	$\text{0}{.\text{0399}}^{**}$ (2.17)
*dg*	$\text{2}{.\text{4809}}^{*}$ (1.78)	${\text{9}.\text{9634}}^{**}$ (2.07)	${\text{9}.\text{2121}}^{*}$ (1.90)	${\text{0}.\text{7303}}^{**}$ (2.16)
*fg*	${-\text{8}.\text{8450}}^{***}$ (−3.56)	${\text{19}.\text{6050}}^{**}$ (2.07)	${\text{25}.\text{1683}}^{***}$ (2.57)	${-\text{4}.\text{0890}}^{***}$ (−5.89)
Controls	Yes	Yes	Yes	Yes
Constant Term	${-\text{0}.\text{4046}}^{**}$ (−2.07)	${\text{2}.\text{9092}}^{***}$ (3.52)	${\text{2}.\text{9871}}^{***}$ (3.36)	${\text{0}.\text{8881}}^{***}$ (10.21)
Individual Fixed Effects	Fixed	Fixed	Fixed	Fixed
Time Fixed Effects	Fixed	Fixed	Fixed	Fixed
Sample Size	1404	1404	1404	1404
${\text{R}}^{\text{2}}$	0.55	0.67	0.69	0.45

The coefficient of domestic agricultural digitalization inputs on agricultural production efficiency is significantly positive and is also positively associated with agricultural DVA, E, and EDVAR. Moreover, agricultural production efficiency has a significantly positive effect on agricultural DVA, E, and EDVAR. To further verify the existence of the mediating effect, this study employs the Bootstrap method for testing. The specific steps are as follows. First, 1,000 random samples are drawn with replacement from the original dataset, and for each resample, a two-way fixed effects regression with robust standard errors is conducted (using the sgmediation2 command). Second, after each regression, the direct, indirect, and total effects are extracted. Finally, based on the 1,000 repeated samples, the mean, Bootstrap standard errors, and confidence intervals of each effect are calculated. Due to space constraints, Bootstrap results are provided in Table E and Table F in S2 Appendix. Results show that the confidence intervals for bs_1 and bs_2 exclude zero, indicating a partial mediation effect. The indirect effects of agricultural production efficiency on DVA, E, and EDVAR are approximately 10.2%, 9.6%, and 11.9%, respectively. These findings suggest that domestic agricultural digitalization inputs enhance agricultural export performance by improving agricultural production efficiency.

Table 9 presents the regression results of the mediation effect model for agricultural trade costs.

**Table 9:** Mediation regression results for agricultural trade costs.

Variables	(1)	(2)	(3)	(4)
	*tc*	*ln (DVA)*	*ln (E)*	*EDVAR*
*tc*		$-\text{0}{.\text{7438}}^{***}$ (−5.82)	$-\text{0}{.\text{8017}}^{***}$ (−6.16)	$\text{0}{.\text{0440}}^{***}$ (3.09)
*dg*	−1.1241 (−0.55)	${\text{9}.\text{3611}}^{**}$ (2.27)	${\text{8}.\text{4070}}^{**}$ (2.06)	${\text{0}.\text{8739}}^{***}$ (2.95)
*fg*	${-\text{5}.\text{5332}}^{*}$ (−1.93)	14.0299 (1.62)	${\text{19}.\text{9047}}^{**}$ (2.27)	${-\text{4}.\text{2688}}^{***}$ (−6.08)
Controls	Yes	Yes	Yes	Yes
Constant Term	${\text{5}.\text{4206}}^{***}$ (16.50)	${\text{6}.\text{8259}}^{***}$ (6.48)	${\text{7}.\text{2320}}^{***}$ (6.61)	${\text{0}.\text{6403}}^{***}$ (5.86)
Individual Fixed Effects	Fixed	Fixed	Fixed	Fixed
Time Fixed Effects	Fixed	Fixed	Fixed	Fixed
Sample Size	1377	1377	1377	1377
${\text{R}}^{\text{2}}$	0.40	0.70	0.72	0.46

The coefficient of foreign agricultural digitalization inputs on agricultural trade costs is significantly negative, while it is positively correlated with agricultural DVA and E, and significantly negatively correlated with EDVAR. Agricultural trade costs have a significantly negative effect on agricultural DVA and E, but a significantly positive effect on EDVAR. To further confirm the mediation effect, a bootstrap test with 1,000 resamples was conducted. Results show that the confidence intervals for both bs_1 and bs_2 exclude zero, indicating a partial mediation effect. The indirect effects of agricultural trade costs on DVA, E, and EDVAR are approximately 22.7%, 18.2%, and 5.4%, respectively. These findings suggest that foreign agricultural digitalization inputs promote agricultural export performance by reducing agricultural trade costs.

## Conclusions and policy implications

This study employs a two-way fixed effects model with country-level clustered robust standard errors to analyze the impact of agricultural digitalization inputs from various sources on agricultural export performance. It also examines the mediating roles of agricultural production efficiency and trade costs. The results show that agricultural digitalization inputs promote economies of scale, reduce information asymmetry, improve supply chain and logistics efficiency, and increase the value-added of agricultural products, thereby enhancing agricultural export performance. Importantly, domestic agricultural digitalization inputs have a stronger and more immediate impact on export performance than foreign inputs, emphasizing the need to strengthen domestic digital capacity. The heterogeneity analysis further reveals that domestic inputs play a more significant role in developing economies, where agricultural digitalization is still in its early stages. This finding underscores the crucial role of endogenous digital innovation in promoting agricultural upgrading and enhancing export competitiveness. Additionally, agricultural GVC position, institutional quality, land resources, and human capital contribute to export performance enhancement. The first three factors exert a stronger influence in developed economies compared to developing ones, implying that developed economies should strengthen their GVC position and leverage strong institutions and efficient land use to boost export performance. Mechanism tests indicate that domestic agricultural digitalization inputs enhance export performance mainly via improved production efficiency, whereas foreign inputs contribute by reducing trade costs. These differentiated mechanisms enrich the literature by offering nuanced evidence on how digitalization affects agricultural trade performance through both productivity and cost channels.

Based on the empirical findings of this study, the following policy recommendations are proposed.

First, promote continuous innovation in digital technologies and accelerate agriculture’s digital transformation. Actively establish platforms for digital technology and asset transactions to facilitate integration across the agricultural sector and drive digital transformation along the entire agricultural value chain. This can be achieved by offering fiscal, tax, and financial policy support, establishing demonstration enterprises and zones for agricultural digitalization, and guiding agricultural enterprises, smallholder farmers, and family farms in accelerating their digital transition. Public-private partnerships should be leveraged to jointly address infrastructure and accessibility gaps in rural areas.

Second, promote self-reliance and resilience in agricultural digitalization to enhance production efficiency. Emphasize domestic agricultural digitalization inputs by strengthening localization and autonomy in agricultural digital transformation. Integrate high-quality domestic resources, consolidate innovation capacity, and address key challenges in agricultural digital technologies. Encourage collaboration between agricultural enterprises and academic institutions for research and development of core digital technologies, including agricultural IoT, sensors, and 5G. Accelerate the transfer of agricultural digital technologies from laboratories to fields, promoting widespread adoption of innovations and thereby improving production efficiency.

Third, optimize the agricultural digital trade environment to reduce trade costs. Enhance agricultural digitalization cooperation with key countries and regions by promoting the signing and implementation of bilateral and multilateral agreements to effectively lower tariff and non-tariff barriers. Assume a leadership role in shaping international digital regulations, advance the liberalization of digital trade, and drive the reform of digital trade rules to reduce costs linked to foreign agricultural digitalization inputs.

Fourth, secure the supply of key production factors to strengthen the foundation for agricultural international competitiveness. Promote agricultural openness and cooperation, accelerate new advantages in agricultural product pricing, information services, and financial insurance, and improve the position in the agricultural GVC. Facilitate the orderly consolidation of land among large-scale farms, agricultural enterprises, and demonstration zones, and further standardize land conservation policies. Enhance the management system for agricultural imports and exports and related laws to create a regulatory environment conducive to private investment. Expand skills training and multi-level talent development to improve the digital literacy of farmers and enterprises, especially smallholders.

Collectively, these policy recommendations highlight that sustainable agricultural export growth relies on a balanced approach that integrates technological innovation, domestic capacity building, trade facilitation, and the optimization of factor endowments. For developing economies, the findings emphasize prioritizing domestic agricultural digitalization inputs while strategically employing foreign technologies to improve efficiency and competitiveness.

In conclusion, this study contributes to the literature by revealing how domestic and foreign agricultural digitalization inputs differentially affect agricultural export performance and by identifying their distinct mechanisms of influence. These insights offer empirical evidence and practical guidance for policymakers seeking to harness digitalization to promote agricultural modernization, enhance export competitiveness, and strengthen global food security. Future research could further examine micro-level digital adoption behaviors and the interactions among digital transformation, environmental sustainability, and inclusive rural development.

## Supporting information

S1 Data Enhancing Sustainable Agricultural Export Performance through Digitalization. https://doi.org/10.6084/m9.figshare.30018103.v2. (XLSX)

S2 Appendix Supplementary Tables for “Enhancing Sustainable Agricultural Export Performance through Digitalization”. https://doi.org/10.6084/m9.figshare.30449426.v1 (PDF)
