# Supplementary material for: Enhancing sustainable agricultural export performance through digitalization: The distinct roles of domestic and foreign inputs
Source: PLoS One. 2025 Dec 30;20(12):e0339692. doi: 10.1371/journal.pone.0339692 (PMC12753084; doi:10.1371/journal.pone.0339692)
Supplement: S2 Appendix — (PDF) [file pone.0339692.s002.pdf]

Supplementary tables for

Enhancing sustainable agricultural export performance through digitalization: The distinct roles of domestic and foreign inputs

Table A. List of 74 Economies

Developed Economies			Developing Economies		
	Code	Country Name		Code	Country Name
2	AUS	Australia	1	ARG	Argentina
3	AUT	Austria	5	BGD	Bangladesh
4	BEL	Belgium	6	BGR	Bulgaria
9	CAN	Canada	7	BRA	Brazil
10	CHE	Switzerland	8	BRN	Brunei Darussalam
17	CYP	Cyprus	11	CHL	Chile
18	CZE	Czechia	12	CHN	China, People's Republic of
19	DEU	Germany	13	CIV	Côte d'Ivoire
20	DNK	Denmark	14	CMR	Cameroon
22	ESP	Spain	15	COL	Colombia
23	EST	Estonia	16	CRI	Costa Rica
24	FIN	Finland	21	EGY	Egypt
25	FRA	France	30	HUN	Hungary
26	GBR	United Kingdom	31	IDN	Indonesia
27	GRC	Greece	32	IND	India
28	HKG	Hong Kong, China	37	JOR	Jordan
29	HRV	Croatia	39	KAZ	Kazakhstan
33	IRL	Ireland	40	KHM	Cambodia
34	ISL	Iceland	42	LAO	Lao (People's Democratic Republic)
35	ISR	Israel	46	MAR	Morocco
36	ITA	Italy	47	MEX	Mexico
38	JPN	Japan	49	MMR	Myanmar
41	KOR	Korea	50	MYS	Malaysia
43	LTU	Lithuania	51	NGA	Nigeria
44	LUX	Luxembourg	55	PAK	Pakistan
45	LVA	Latvia	56	PER	Peru
48	MLT	Malta	57	PHL	Philippines
52	NLD	Netherlands	58	POL	Poland
53	NOR	Norway	60	ROU	Romania
54	NZL	New Zealand	61	RUS	Russian Federation
59	PRT	Portugal	62	SAU	Saudi Arabia
64	SGP	Singapore	63	SEN	Senegal
65	SVK	Slovak Republic	68	THA	Thailand
66	SVN	Slovenia	69	TUN	Tunisia
67	SWE	Sweden	70	TUR	Türkiye
72	USA	United States	71	UKR	Ukraine
			73	VNM	Viet Nam
			74	ZAF	South Africa

Table B. Correlation Coefficient Test Results

	dg	fg	GP	te	rq	hc	lr	lp	tc
dg	1.000								
fg	0.3237^{***}	1.000							
GP	-0.0573^{**}	-0.2914^{***}	1.000						
te	0.2758^{***}	0.2760^{***}	-0.1745^{***}	1.000					
rq	0.4244^{***}	0.5313^{***}	-0.3868^{***}	0.5312^{***}	1.000				
hc	0.3109^{***}	0.3562^{***}	-0.4011^{***}	0.5725^{***}	0.7628^{***}	1.000			
lr	-0.2228^{***}	-0.1792^{***}	0.0120	0.0474^{*}	-0.1426^{***}	-0.0567^{**}	1.000		
pe	0.1129^{***}	0.1370^{***}	-0.1594^{***}	0.3350^{***}	0.3276^{***}	0.3967^{***}	0.0281	1.000	
tc	-0.1270^{***}	-0.2381^{***}	0.2343^{***}	-0.4133^{***}	-0.4802^{***}	-0.4677^{***}	-0.1856^{***}	-0.2686^{***}	1.000
VIF	1.300	1.440	1.260	1.610	3.190	2.750	1.180	1.200	1.510
1/VIF	0.771	0.697	0.795	0.622	0.313	0.364	0.851	0.833	0.663

Note: ***; **; * indicate significance at the 1%, 5%, and 10% confidence levels, respectively, with the t-statistics shown in parentheses. The same applies to the following text.

Table C. Robustness Test Results for the Dependent Variable (EDVAR)

	Substitution of Dependent Variables	Substitution of Independent Variables	Winsorization Treatment	Multidimensional Clustering
dg	0.4731<br>(1.20)	0.0005<br>(0.01)	0.9141^{**}<br>(2.08)	0.8292^{**}<br>(2.79)
fg	-4.2797^{***}<br>(-4.69)	-4.1232^{***}<br>(-4.42)	-4.6046^{***}<br>(-5.88)	-4.4416^{***}<br>(-5.74)
Controls	Yes	Yes	Yes	Yes
Individual Fixed Effects	Fixed	Fixed	Fixed	Fixed
Time Fixed Effects	Fixed	Fixed	Fixed	Fixed
Sample Size	1168	1168	1404	1404
R^2	0.44	0.43	0.45	0.26

Table D. Results of Sequential Exclusion of High-Leverage Countries

Country	Variables	Ln(*DVA*)	Ln*E*	*EDVAR*
USA	*dg*	11.2303**	10.3268**	0.8333**
		(2.466)	(2.256)	(2.544)
	fg	15.4628*	21.5525**	−4.4379***
		(1.682)	(2.294)	(-6.046)
BRA	*dg*	11.8858***	10.9571**	0.8385**
		(2.638)	(2.411)	(2.559)
	fg	15.0097*	21.1230**	−4.4444***
		(1.667)	(2.291)	(-6.026)
CAN	*dg*	11.1065**	10.2077**	0.8274**
		(2.441)	(2.233)	(2.549)
	fg	15.6553*	21.7284**	−4.4216***
		(1.694)	(2.299)	(-6.017)
FRA	*dg*	10.7601**	9.8406**	0.8418**
		(2.352)	(2.142)	(2.595)
	fg	15.8984*	22.0112**	−4.452***
		(1.726)	(2.337)	(-6.061)
NLD	*dg*	10.3293**	9.4520**	0.8147**
		(2.219)	(2.023)	(2.485)
	fg	15.9207*	22.0093**	−4.4361***
		(1.725)	(2.332)	(-6.043)
ESP	*dg*	11.0855**	10.1839**	0.8292**
		(2.429)	(2.221)	(2.558)
	fg	15.4873*	21.5770**	−4.4379***
		(1.680)	(2.290)	(-6.038)
CHN	*dg*	11.0013**	10.0941**	0.8336**
		(2.410)	(2.201)	(2.568)
	fg	16.2109*	22.3662**	−4.4926***
		(1.745)	(2.352)	(-6.011)
AUS	*dg*	11.0821**	10.1917**	0.8222**
		(2.432)	(2.228)	(2.536)
	fg	15.4035*	21.4919**	−4.4347***
		(1.671)	(2.281)	(-6.034)
RUS	*dg*	10.4971**	9.6144**	0.8181**
		(2.304)	(2.102)	(2.501)
	fg	16.3073*	22.3804**	−4.4258***
		(1.773)	(2.378)	(-6.037)
ARG	*dg*	11.5295**	10.6546**	0.8154**
		(2.529)	(2.329)	(2.487)
	fg	15.4390*	21.5332**	−4.4394***
		(1.687)	(2.300)	(-6.072)

Table E. Bootstrap Mediation Effect Decomposition of Domestic Agricultural Digitalization Inputs

Dependent variable	Mediating variable	effect	Observed Coef.	Std. Err.	z	P>|z|
ln*(DVA)*	*lp*	Indirect effect	1.1270	0.4913	2.29	0.022
		Direct effect	9.9633	3.1920	3.12	0.002
		Total effect	11.0904	3.1252	3.55	0.000
ln*(E)*	*lp*	Indirect effect	0..9773	0.4861	2.01	0.044
		Direct effect	9.2121	3.3811	2.72	0.006
		Total effect	10.1894	3.3389	3.05	0.002
EDVAR	*lp*	Indirect effect	0.0989	0.0354	2.79	0.005
		Direct effect	0.7303	0.1803	4.05	0.000
		Total effect	0.8292	0.1842	4.50	0.000

Table F. Bootstrap Mediation Effect Decomposition of Foreign Agricultural Digitalization Inputs

Dependent variable	Mediating variable	effect	Observed Coef.	Std. Err.	z	P>|z|
ln*(DVA)*	*tc*	Indirect effect	4.1158	1.4357	2.87	0.004
		Direct effect	14.0299	4.8053	2.92	0.004
		Total effect	18.1456	4.5212	4.01	0.000
ln*(E)*	*tc*	Indirect effect	4.4360	1.4843	2.99	0.003
		Direct effect	19.9047	4.6111	4.32	0.000
		Total effect	24.3408	4.4077	5.52	0.000
EDVAR	*tc*	Indirect effect	-0.2433	0.0868	-2.80	0.005
		Direct effect	-4.2687	0.3897	-10.96	0.000
		Total effect	-4.5120	0.4080	-11.06	0.000
